# Hidden Adaptations: Ultrasound Evidence of Intrinsic Foot and Tendon Changes in Basketball Players with Hallux Limitus

**DOI:** 10.3390/jcm14228154

**Published:** 2025-11-17

**Authors:** Samuel Eloy Gutiérrez-Torre, Nerea Molina-Hernández, Álvaro García-Vázquez, César Calvo-Lobo, David Rodríguez-Sanz, Ricardo Becerro-de-Bengoa-Vallejo

**Affiliations:** 1 Facultad de Enfermería, Fisioterapia y Podología, Universidad Complutense de Madrid, 28040 Madrid, Spain; neremoli@ucm.es (N.M.-H.); davidrodriguezsanz@ucm.es (D.R.-S.); ribebeva@ucm.es (R.B.-d.-B.-V.); 2 Facultad de Ciencias de la Salud, Universidad Rey Juan Carlos, 28922 Alcorcón, Spain; alvgar25@ucm.es or a.garciava.2022@alumnos.urjc.es

**Keywords:** hallux limitus, ultrasound, foot, basketball players

## Abstract

**Background:** Hallux limitus (HL) is a restriction of first metatarsophalangeal joint dorsiflexion, commonly linked to foot biomechanics-related disorders or trauma, increasing sports injury risk. It involves plantar fascia tension, compensations, and tendon adaptations. Rehabilitative ultrasound imaging (RUSI) accurately assesses musculoskeletal changes, supporting physiotherapy evaluation and the study of HL-related structural adaptations. **Objectives:** Comparing the thickness and cross-sectional area (CSA) of flexor hallucis brevis (FHB), flexor digitorum brevis (FDB), abductor hallucis (AbH), and quadratus plantae (QP) muscles, as well as the thickness of the plantar fascia (PF), Achilles tendon (AT), and plantar calcaneal fat pad (CFP), between participants with and without HL. **Methods:** Case–control study included 80 basketball players recruited from semi-professional teams by consecutive non-probabilistic sampling. Participants were divided into two groups: an HL group (n = 40) and a healthy group (n = 40). Musculotendinous parameters were assessed using RUSI. **Results:** The FDB, FHB, AB, and QP showed significant reductions in thickness and CSA at rest and at contraction in the HL group. PF thickness increased in participants with HL, while CFP thickness decreased significantly. Significance was established at (*p* < 0.05). **Conclusions:** HL participants exhibited reduced muscle size and CSA, increased PF, and lower CFP thicknesses, indicating adaptive tissue alterations.

## 1. Background

Hallux limitus (HL) was recently defined as restricted dorsiflexion mobility in the first metatarsophalangeal joint (MTPJ), with a range of motion of less than 65° [1]. Hallux dorsiflexion mobility can be evaluated in two basic ways: (1) under weight-bearing conditions and (2) without bearing weight. Several techniques exist for evaluating the mobility of the great toe using goniometry, with normal ranges of motion ranging historically from 37° to 82° [2,3,4]. Its etiology is multifactorial, with foot posture and function playing a fundamental role in its development [5]. Repeated trauma, hypermobility of the first radius, as well as its dorsiflexion, or the presence of flat feet may also be involved in its development, among other causes [6,7]. Reduced mobility in this joint can alter the loads and pressures it receives [8], thus increasing the risk of injury, especially in high-impact sports such as basketball [9]. 

Viehöfer et al. [10] described how increased tension in the plantar fascia (PF) could be involved in the development of HL. In turn, Maceira & Monteagudo [11] describe the mechanisms by which anatomical compensations could occur at the level of the plantar Achilles-calcaneal system, in the extrinsic musculature of the foot, or even at the knee. In relation to this, it has been demonstrated how adaptations occur at the muscle-tendon level in patients with Achilles tendinopathy in response to load [12,13].

Rehabilitation ultrasound imaging (RUSI) has proven to be a valid tool for quantifying both body cross-sectional area (CSA) and the thickness of muscles and connective tissue in musculoskeletal disorders, which is useful in physiotherapy assessment [14,15]. In the case of the intrinsic foot muscles (IFM) and PF, parameters such as CSA and thickness allow relevant associations to be established between foot function and certain clinical conditions, such as flat feet or Achilles tendinopathy [16,17]. 

Therefore, it is interesting to explore the role of foot and ankle structures and their relationship with HL, as they have not been sufficiently investigated to date. The aim of this study was to compare and quantify, using RUSI, the thickness and CSA of the flexor hallucis brevis (FHB), flexor digitorum brevis (FDB), abductor hallucis (AbH), and quadratus plantae (QP) muscles, as well as the PF, Achilles tendon (AT), and calcaneal fat pad (CFP) in professional basketball players with and without HL.

## 2. Methods

### 2.1. Study Design

A case–control study was conducted between December 2022 and May 2023, designed in accordance with the criteria of the STROBE (Strengthening the Reporting of Observational Studies in Epidemiology) guidelines [18,19] to compare physical factors in athletes with hallux limitus and healthy athletes.

### 2.2. Ethics

The study was approved by the Ethics Committee of Hospital Clínico San Carlos (22/671-E) and complies with Organic Law 3/2018 on data protection, Order SAS 3470/2009, the Declaration of Helsinki (2013) and Law 14/2007 on biomedical research. Participants were coded with numbers from 0 to 80, ensuring their confidentiality and anonymity. Before starting, the principal investigator explained the study procedure in the information leaflet and informed consent form. All participants signed the informed consent form, although they could sign a revocation format any time during the study if they wished.

### 2.3. Patient Selection and Sample Size Estimation

Recruitment for the study was carried out using consecutive non-probabilistic sampling at the sports facilities of the following basketball clubs, Real Canoe N.C., Óvila Club de Basket, Club Bazu Baloncesto Azudense, Club Deportivo Baloncesto Segovia, and Club de Baloncesto Uros de Rivas, all of which belonged to the Spanish basketball leagues Primera Nacional Española, Liga de Baloncesto Aficionado Española, and Liga de Baloncesto Española.

The case group included male participants aged between 18 and 65 years old diagnosed with bilateral hallux limitus using Buell’s non-weight-bearing goniometric technique [1,2] by a research assistant, with no alterations in the regions evaluated and with the same level of athleticism as the participants in the control group.

The exclusion criteria were pathology at the time of assessment affecting the lower limbs and the structures evaluated, preventing the various measurements from being taken; acute injury to the ankle and/or any other assessed region in the 3 months prior to entry into the study, resulting in the loss of at least one day of usual physical activity; as well as subjects with any systemic disease or infection [20] or fracture surgery [21].

G*Power 3.1.9.2 software (G*Power^®^, University of Dusseldorf, Germany) was used to calculate the sample size based on the measurement of the primary outcome of left PF thickness (cm) at rest from a pilot study (n = 26). PF thickness was chosen due to the important role of this structure in the potential development of HL [10,11]. Furthermore, because the study assessed symmetrical variables, it was necessary to select one of the two sides for the sample size calculation. Therefore, the preliminary pilot study was divided into two groups (mean ± SD): 13 subjects with HL (0.453 ± 0.145) and 13 individuals for the healthy group (0.380 ± 0.069). To calculate the power, an α error of 0.05, an effect size of 0.640, a power of 0.80, and a two-tailed hypothesis were used to calculate the sample size. Finally, a total sample of 80 individuals (40 per group) was calculated.

### 2.4. Ultrasound Imaging

The main objective of the experiment was to determine the morphology (GE Healthcare, Wauwatosa, WI, USA) of the muscles FHB, FDB, AbH, and QP, as well as the non-muscular structures of PF, CFP, and AT, using a Mindray Z6 ultrasound scanner (Shenzhen Mindray Bio-Medical Electronics, Nansham, China) and a linear probe with a frequency range of 5 to 10 MHz and a 38 mm probe foot (type 7 L4P; 38 mm probe foot), using B-mode.

Both the ultrasound evaluations and tests based on the Foot Posture Index (FPI) were performed by the same evaluator (SE-G-T), a podiatrist and physiotherapist with 5 years of experience in performing the RUSI technique. The evaluator did not know which group the participants belonged to before performing the evaluation. Prior to image acquisition, a personal interview was conducted with each player, in which sociodemographic and personal variables were collected, and the group to which the player belonged was defined. This process was carried out by an assistant researcher, with an average duration of 5–10 min within the total study period (30–45 min in total).

The parameters of frequency, depth, gain, and focus position were adjusted according to each subject and each evaluated structure in order to obtain the highest possible image quality [22].

**Figure 1:**
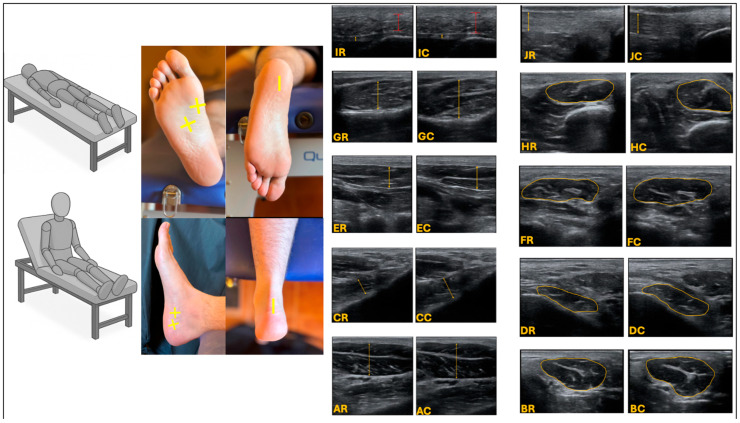
Ultrasound imaging thickness, and CSA for the AbH, FHB, FDB, QP, PF, CFP and AT. Abbreviation: AR, AbH thickness in resting state; AC, AbH thickness in contraction; BR, AbH CSA in resting state; BC, AbH CSA in contraction; CR, OP thickness in resting state: CC, OP thickness in contraction; DR, QP CSA in resting state; DC, QP CSA in contraction; ER, FDB thickness in resting state; EC, FDB thickness in contraction; FR, FDB CSA in resting state; FC, FDB CSA in contraction; GR, FHB thickness in resting state; GC, FHB thickness in contraction; HR, FHB CSA in resting state: HC, FHB CSA in contraction; IR. PF (orange colour) and CFP (red colour) thickness in resting state: IC, PF (orange colour), and CFP (red colour) thickness in contraction; JR, AT thickness in resting state; JC, AT thickness in contraction.

The ultrasound analysis of the IFM followed the protocol of Mickle et al. [23] and Crofts et al. [16]. In the case of Croft et al., the inter-observer reliability ranged from very good to excellent for both the CSA of the AbH (ICC = 0.91), FDB (ICC = 0.98), and FHB (ICC = 0.95) muscles and for the thicknesses of the same muscles: AbH (ICC = 0.92), FDB (ICC = 0.96), and FHB (ICC = 0.97). Similarly, in the case of Mickle et al., inter-rater reliability was also excellent for both the CSA of the QP muscle (ICC = 0.99) and for the thickness of the same QP muscle (ICC = 0.97). The evaluation of the PF followed the protocol of Skovdal Rathleff et al. [24]; the PF’s intra- and inter-examiner reliability (ICC range 0.77–0.82) was excellent. The AT was assessed using the protocol developed by del Baño-Aledo et al. [25] who found to have excellent intra- and inter-examiner reliability (ICC range 0.98–0.99). The protocol of Lopez-Lopez et al. [26], was followed for the evaluation of the CFP, which had excellent inter- and intra-examiner reliability (ICC = 0.93). The mentioned protocols are as follows (Figure 1):

FHB: Each patient was assessed in a prone position on a physiotherapy table. To measure resting muscle thickness, an ultrasound transducer was placed longitudinally under the first metatarsal (slightly posterolaterally oblique). A proximal scan was then performed to locate the thickest portion of the muscle belly, distal to the base of the first metatarsal. Next, to measure the resting CSA, the ultrasound transducer was rotated 90 degrees. Images were then acquired during contraction, repeating the protocol to obtain both the thickness and CSA images, where each participant was asked to voluntarily contract the structure being evaluated.

AbH: Each patient was evaluated in a supine position, with slight external rotation of the hip and slight flexion of the knee, on a physiotherapy table. To measure resting muscle thickness, the ultrasound transducer was placed on the medial tuberosity of the calcaneus, in the direction of the scaphoid tubercle. Normally, the thickest area is located 1–2 cm proximal to the scaphoid tubercle. A transversal image was also taken, rotating the ultrasound probe to determine the resting CSA. Images were then acquired during contraction, repeating the protocol to obtain both the thickness and CSA images, where each participant was asked to voluntarily contract the structure being evaluated.

QP: Each patient was evaluated in a prone position on a physiotherapy table. The QP was located in the depths of the FDB. Next, we located the talocalcaneal-scaphoid joint and, using the longitudinal probe, aligned it in the direction of the muscle fibers, looking for the thickest area in the muscle belly, which is usually found proximal to the elastic ligament. We then measured the resting thickness in the longitudinal section and the resting CSA in the transverse section. Images were then acquired during contraction, repeating the protocol to also obtain both the thickness and CSA images, where each participant was asked to voluntarily contract the structure being evaluated.

FDB: Each patient was assessed in a prone position on a physiotherapy table. A line was drawn between the medial calcaneal tubercle and the third toe. The probe was placed longitudinally along this line, extending from the insertion in the calcaneus, and a distal scan was performed to locate the thickest area of the muscle belly, before dividing it into four fascicles. The image was taken longitudinally to determine the resting thickness of the longitudinal section and transversely to determine the resting CSA. After succeeding, images were then acquired during contraction, repeating the protocol to obtain both the thickness and CSA images where each participant was asked to voluntarily contract the structure being evaluated.

AT: Each patient was assessed in a prone position on a physiotherapy table with their feet hanging down. To determine the resting thickness, measurements were taken on the longitudinal axis 3 cm proximal to the tendon insertion in the calcaneus bone. Subsequently, images were then acquired during contraction, repeating the protocol to obtain the thickness wherein the participant voluntarily contracted the structure being evaluated. However, due to this structure lacking contractile capacity, participants were asked to perform a voluntary plantarflexion contraction of their ankle.

PF: Each patient was assessed in a prone position on a physiotherapy table with their feet hanging down. To determine the resting thickness, the ultrasound transducer was placed on the calcaneus, over the line between the medial calcaneal tubercle and the great toe on the sole of the foot. Next, images were acquired during contraction, repeating the protocol to obtain the thickness while the participant was voluntarily contracting the structure being evaluated. However, due to this structure lacking contractile capacity, participants were asked to perform a voluntary plantarflexion contraction of their toes.

CFP: The patient was placed in a prone position on a stretcher where the resting thickness measurements were taken with the ankle flexed at 90°. The calcaneal tuberosity was palpated, and the transducer was placed. No pressure was applied so as not to deform the CFP. The transducer was oriented along the longitudinal axis, towards the 2nd metatarsal. Next, images were then acquired during contraction, repeating the protocol to obtain the thickness wherein the participant voluntarily contracted the structure being evaluated; however, due to this structure lacking contractile capacity, participants were asked to perform a voluntary plantarflexion contraction of their toes.

### 2.5. Image Measurement

Three images were collected for each moment (rest/contraction) and for each side (right/left), calculating the average of the three measurements [27]. ImageJ version 2.0 software (National Institutes of Health, Bethesda, MD, USA) was used to measure the different structures. In addition, muscle contraction capacity was determined for both CSA and thickness measurements using the activation ratio (AR) (AR = active contraction thickness/resting thickness) [28]. 

### 2.6. Data Analysis

Statistical analysis was performed using SPSS v.22.0 (IBM, Armonk, NY: IBM Corp, Westchester County, NY, USA). First, the Kolmogorov–Smirnov test was performed to assess the normal distribution of the data. Second, a descriptive analysis was performed for all individuals. Finally, a comparative analysis was performed between the HL group and the control group, as well as a comparative analysis between both feet. The mean and standard deviation (SD) were calculated using Student’s *t*-test for independent samples, and the median and interquartile range (IQR) were calculated using the Mann–Whitney U test for parametric and non-parametric data, respectively. In addition, Levene’s test was used to assess the equality of variances (parametric data). Furthermore, Pearson and Spearman’s rank correlations were calculated to determine the associations between the right foot and left foot variables in order to compare the variables between these feet using paired-samples Student *t*-test for paired samples and Wilcoxon test for paired samples, depending on the data distribution. Likewise, a chi-square test was used to compare the differences between the jumping leg and the group. To interpret the effect size, Cohen’s d statistic (d = 0.200 = small; d = 0.500 = medium; d = 0.800 = large) [29] and Rosenthal’s r (r ≈ 0.100 = small; r ≈ 0.300 = medium; r ≈ 0.500 = large) were used for comparisons with *p* < 0.05 [30].

Additionally, a multiple linear regression model was performed by the stepwise method to predict the main dependent variable of this study based on the descriptive variables and HL presence. The quality of adjustment was carried out by the R^2^ coefficient. *p*_in_ and *p*_out_ values were considered as 0.05 and 0.10, respectively. The main descriptive variable was the thickness of the plantar fascia at resting state (according to the provided sample size calculation previously described). The independent variables were the presence of HL and the descriptive data (age, weight, height, BMI, etc.).

## 3. Outcomes

For sociodemographic variables (Table 1), the sample proved to be homogeneous, as no statistically significant differences were found between the groups. Regarding the main results of the study, in relation to IFM (Table 2), FHB CSA measurements showed statistically significant differences during contraction on both sides (right (R) and left (L)) (R, *p* = 0.007, d = 0.620; L, *p* = 0.002, d = 0.717), with larger values in the healthy group. For the same structure, there were statistically significant differences in favor of the healthy group in the thicknesses on both sides in a resting state (R, *p* = 0.045, d = 0.383; L, *p* = 0.022, d = 0.457) and contraction (R, *p* ≤ 0.001, d = 1118; L, *p ≤* 0.001, r = −0.635). The AR differences for this variable on both sides, both in thickness (R, *p ≤* 0.001, r = −0.625; L, *p ≤* 0001, r = −0.702) and CSA (R, *p ≤* 0.001, r = −0.661; L, *p ≤* 0.001, d = 1.324), were statistically significant and larger in the healthy group. The measurement of AbH thickness showed statistically significant differences in the healthy group for the variable of thickness on the left side during contraction (*p* = 0.009, d = 0.600). In turn, AR differences for this variable on both sides were evident in the healthy group, both for thickness (R, *p ≤* 0.001, r = −0.430; L, *p ≤* 0.001, r = −0.495) and CSA (R, *p ≤* 0.001, d = 1.156; L, *p ≤* 0.001, d = 1.344). For the FDB structure, statistically significant differences were obtained for all variables in favor of the healthy group in thickness both in the relaxed state (R, *p ≤* 0.001, r = –0.378; L, *p ≤* 0.001, r = −0.426), as well as during contraction (R, *p ≤* 0.001, d = 1.470; L, *p ≤* 0001, r = −0.495). The same was true for CSA at rest (R, *p* = 0.007, d = 0.619; L, *p* = 0.004, d = 0.610), and during contraction (R, *p ≤* 0.001, d = 0.832; L, *p* = 0.002, d = 0.711). The AR differences in the FDB variable on both sides, both in thickness (R, *p ≤* 0.001, r = −0.521; L, *p ≤* 0.001, r = −0.446), and CSA (R, *p ≤* 0.001, r = −0.394; L, *p* = 0.004, r = –0.320), were statistically significant, with higher values in the healthy group. For the QP variable, statistically significant differences were detected, with the healthy group having higher values for the variables of left thickness and right CSA during contraction (L, *p ≤* 0.001, d = 0.717; R, *p* = 0.033, d = 0.419). The AR differences for the QP variable on both sides, both in thickness (R, *p ≤* 0.001, r = −0.721; L, *p ≤* 0.001, r = −0.751) and CSA (R, *p ≤* 0.001, d = 1.136; L, *p* = 0 < 0.001, r = −0.450), were statistically significant between the HL group compared to the healthy group. For the PF variable, statistically significant differences were observed in bilateral resting thicknesses (R, *p ≤* 0.001, r = −0.564; L, *p ≤* 0.001, r = −0.391), with higher values in the case group. In contrast, the differences found in the AR variables for the PF indicated larger AR values in the control group bilaterally (R, *p ≤* 0.001, r = −0.645; L, *p ≤* 0.001, r = −0.681). For the CFP variable, statistically significant differences were observed for the control group, both in thickness at rest and during contraction bilaterally (R, *p* = 0.003, d = 0.684; L, *p* = 0.009, d = 0.599) (R, *p* = 0.003, d = 0.689; L, *p* = 0.001, r = −0.366). Finally, statistically significant differences were found in the RA for the TA variable on the left side (L, *p* = 0.036, r = −0.235). There were no statistical differences in the other variables (Table 3).

For the correlations between the variables of both feet (Table 4), statistically significant differences were found for all of them (*p ≤* 0.001), with moderate to strong positive correlations (r > 0.500, ρ > 0.400).

For the comparisons between both feet (Table 5), statistically significant differences were only obtained for the thicknesses of the CFP (*p* = 0.044, d= −0.229) and FDB (*p* = 0.008, d= −0.306) variables at rest, as well as for the thicknesses of the contraction variables FHB (*p* = 0.033, d = −0.243) and AT (*p* = 0.041, d = −0.232). The rest of the variables did not show statistically significant differences.

Furthermore, a linear regression model (*R*^2^ = 0.388; F_1,78_ = 13.858; β = 0.075; *p* ˂ 0.001) predicted the thickness of the plantar fascia in a resting state based only on the presence of HL. The rest of the independent variables (age, height, BMI, etc.) were excluded from the linear regression model and did not predict or influence the results of this study regarding the thickness of the plantar fascia in a resting state as the main outcome measurement, according to the *p*_in_ and *p*_out_ values.

## 4. Discussion

This could be considered the first study whose main objective was to determine both passive and dynamic differences in various IFM structures between basketball players with and without HL. The IFM plays a fundamental role in foot and ankle control, especially in patients with flat feet [31,32,33], and/or forefoot pathology [34].

The FDB and AbH muscles have elastic musculotendinous units, which are capable of storing energy, while the IFM contracts quasi-isometrically [35,36]. The FDB and AbH muscles are known to be closely associated with the medial longitudinal arch of the foot [32] and are capable of adapting their mechanical properties to facilitate the functioning of tendon structures, contributing to the support of the plantar arch, as well as generating propulsive or decelerative power [37]. This could be the reason why our study found statistically significant differences in all FDB variables as a main outcome, with a reduction in both thickness and CSA, as well as in AR in participants with HL; or it could also be due to the deficit in IFM function and contractility in the presence of forefoot pathology, specifically in the first radius [38]. Furthermore, deficits in the functioning of these muscles could be directly related to the viscoelastic capacities of tendon structures such as the PF or AT [39]. In our case, we found an increase in thickness at rest in favor of the HL group for the PF, our primary variable, coinciding with previous studies by Lobo et al. [40] conducted on patients with hallux valgus. This could also be in line with Cook et al. in relation to the etiology of tendinopathies [41]. Even so, it is known that the PF plays a fundamental role in the development of HL [10], suggesting its involvement in these modifications. Furthermore, our results show a reduction in CFP thickness, supporting the results obtained in the literature by Romero-Morales et al. [17] in participants with Achilles tendinopathy. This reaffirms the close relationship between these structures.

Additionally, it is known that the QP assists the flexion of the lesser toes through its connection to the flexor digitorum longus (FDL). However, Edama et al. [42] demonstrated that its main function is related to the lateral traction of the FDL and flexor hallucis longus (FHL) tendons. This could be related to the increase in thickness and CSA during contraction in our study. Even so, we have not been able to verify why, in certain muscles, a decrease was observed in thickness, and in others, in CSA. We speculate that this could be due to the predominant use of the left leg for jumping in the participants in this study, which would require a more prominent angle of pennation in the musculature and, therefore, a larger CSA to meet these demands [43,44]. Furthermore, in certain structures such as the FHB, AT, or CFP, this discrepancy in thickness can be observed, favoring the jumping leg side (Table 5).

Likewise, we found a significant reduction in both thickness and CSA of the FHB structure in the case group. This may be due to the mechanical deficit suffered by the first MTFJ in the presence of restricted mobility, given that this favors lower muscle activity [45], which could be due to lower muscle recruitment and a smaller variation in the volumes of that area. Another theory could be related to the trade-off effect in relation to the angle of pennation, range of motion (ROM), and contraction speed. It is known that muscles with greater pennation angles are capable of applying a large amount of force at the expense of decreasing contraction speed and limiting ROM [46,47], which could explain the deficit in muscle size in this region. These same findings are reinforced by the results of Romero-Morales et al. [48], who found similar results in participants with Achilles tendinopathy, where the plantar Achilles–calcaneus system plays a fundamental role due to its connection with the PF [10]. 

Regarding AR, all IFM variables in participants with HL showed a statistically significant reduction. This could be due to limited motor control of the IFM or even proprioceptive problems related to muscle weakness in this muscle group [49,50,51]. 

### Limitations

This study had multiple limitations. For one, all data were collected from male Spanish semi-professional basketball players, which limits generalizability to women, other sports, or the general population; therefore, future research should include such parameters to broaden the external validity of the data. For another, muscle strength was not directly assessed using, for example, dynamometry or pennation angles; we speculate that participants with HL would have lower strength values as well as altered pennation angles compared to the group without HL, as shown by García-García et al. [19] in handball players with CAI compared to healthy players. Furthermore, future research should focus more extensively on the jumping leg, given its significant role in this sport. Likewise, it would be advisable to investigate in the future the influence of confounding factors that may be related to the changes observed in this study as well as make comparisons more than just parallel tests to avoid type I error when analyzing other covariates. Finally, the evaluations of this study were conducted by a single observer, so future investigations should include at least two observers to analyze image reliability and inter-observer reproducibility.

## 5. Conclusions

The thicknesses and CSAs of the FDB, FHB, AbH, and QP muscles were reduced in participants with HL. Participants with HL showed an increase in PF thickness, as well as a reduction in CFP thickness. Participants with HL had lower ARs compared to healthy participants.

## Figures and Tables

**Table 1:** Sociodemographic data and Foot Postural Index of the sample.

Measurement	Control Group (n = 40)	Case Group (n = 40)	*p*-Value Cases vs. Controls
Weight, kg	87.11 ± 15.49 ^a^	88.60 ± 12.22 ^a^	0.317 ^b^
Height, cm	189.53 ± 7.95 ^a^	191.35 ± 7.91 ^a^	0.307 ^b^
Age, y	22.50 ± 6.00 ^c^	23.00 ± 8.00 ^c^	0.159 ^d^
IPAC, METS/min/week	7472.20 ± 2809.82 ^a^	7319.25 ± 3648.00 ^c^	0.866 ^d^
Physical Activity, y	15.13 ± 4.28 ^a^	15.53 ± 5.55 ^a^	0.719 ^b^
BMI, kg/m^2^	24.20 ± 3.10 ^c^	24.09 ± 1.97 ^a^	0.821 ^d^
Right foot FPI	1.12 ± 5.25 ^a^	1.00 ± 7.00 ^c^	0.973 ^d^
Left foot FPI	1.65 ± 5.68 ^a^	1.00 ± 7.75 ^c^	0.524 ^d^
Jumping Leg, right/left	6/34	5/35	0.745 ^e^

Abbreviations: IPAC, (International Physical Activity Questionnaire); BMI, Body Mass Index; FPI, Foot Postural Index. ^a^ Mean ± standard deviation (SD). ^b^ Student’s *t*-test for independent samples. ^c^ Median ± interquartile range (IR). ^d^ Wilcoxon Mann–Whitney *U* test. ^e^ χ^2^ Chi-square test.

**Table 2:** Ultrasound imaging measurements and activation ranges of IFM.

Measurement	Control Group (n = 40)	Case Group (n = 40)	*p*-Value	d-Cohen	r (Rosenthal)
Right side					
Cross-Sectional Area					
FHB (resting), (cm^2^)	2.762 ± 0.764 (0.891–3.996) ^a^	2.672 ± 0.828 (1.158–4.445) ^a^	0.611 ^b^	0.114	-
FHB (contracted) (cm^2^)	3.306 ± 0.815 (0.943–4.599) ^a^	2.778 ± 0.883 (0.997–4.560) ^a^	**0.007 ^b^**	0.620	-
AbH (resting) (cm^2^)	2.411 ± 0.530 (1.152–3.598) ^a^	2.618 ± 0.564 (0.888–3.950) ^a^	0.095 ^b^	−0.378	-
AbH (contracted) (cm^2^)	2.740 ± 0.618 (1.509–4.464) ^a^	2.727 ± 0.631 (1.065–4.108) ^a^	0.926 ^b^	0.021	-
FDB (resting) (cm^2^)	2.521 ± 0.571 (1.758–4.185) ^a^	2.168 ± 0.569 (1.343–3.580) ^a^	**0.007 ^b^**	0.619	-
FDB (contracted) (cm^2^)	2.750 ± 0.572 (1.867–4.411) ^a^	2.245 ± 0.638 (1.390–3.733) ^a^	**<0.001 ^b^**	0.832	-
QP (resting) (cm^2^)	1.710 ± 0.443 (0.745–2.783) ^a^	1.762 ± 0.371 (1.144–2.654) ^a^	0.572 ^b^	−0.127	-
QP (contracted) (cm^2^)	2.063 ± 0.610 (1.005–3.697) ^a^	1.851 ± 0.374 (1.181–2.688) ^a^	**0.033 ^b^**	0.419	-
AR CSA FHB R	1.175 ± 0.123 (1.059–2.200) ^c^	1.021 ± 0.093 (0.861–1.359) ^c^	**<0.001 ^d^**	-	−0.661
AR CSA AbH R	1.140 ± 0.094 (0.966–1.429) ^a^	1.042 ± 0.073 (0.846–1.247) ^a^	**<0.001 ^b^**	1.156	-
AR CSA FDB R	1.113 ± 0.107 (0.916–1.411) ^a^	1.022 ± 0.091 (0.497–1.389) ^c^	**<0.001 ^d^**	-	−0.394
AR CSA QP R	1.206 ± 0.174 (0.947–1.755) ^a^	1.053 ± 0.075 (0.880–1.272) ^a^	**<0.001 ^b^**	1.136	-
Thickness					
FHB (resting) (cm)	1.221 ± 0.197 (0.711–1.671) ^a^	1.142 ± 0.213 (0.654–1.567) ^a^	**0045 ^b^**	0.383	-
FHB (contracted) (cm)	1.480 ± 0.214 (1.040–1.891) ^a^	1.229 ± 0.234 (0.706–1.621) ^a^	**<0.001 ^b^**	1.118	-
AbH (resting) (cm)	1.073 ± 0.137 (0.758–1.359) ^a^	1.105 ± 0.328 (0.835–1.695) ^c^	0.246 ^d^	-	−0.130
AbH (contracted) (cm)	1.210 ± 0.170 (0.758–1.595) ^a^	1.153 ± 0.244 (0.920–1.785) ^c^	0.384 ^d^	-	−0.097
FDB (resting) (cm)	0.949 ± 0.208 (0.736–1.262) ^c^	0.853 ± 0.157 (0.545–1.224) ^a^	**<0.001 ^d^**	-	−0.378
FDB (contracted) (cm)	1.138 ± 0.169 (0.823–1.503) ^a^	0.899 ± 0.155 (0.661–1.188) ^a^	**<0.001 ^b^**	1.470	-
QP (resting) (cm)	1.033 ± 0.245 (0.478–1.251) ^c^	1.056 ± 0.214 (0.765–1.538) ^a^	0.538 ^d^	-	−0.069
QP (contracted) (cm)	1.274 ± 0.149 (0.763–1.463) ^c^	1.100 ± 0.211 (0.808–1.707) ^a^	0.106 ^d^	-	−0.181
AR THICKNESS FHB R	1.220 ± 0.104 (1.063–1.463) ^a^	1.078 ± 0.085 (0.905–1.294) ^a^	**<0.001 ^d^**	-	−0.625
AR THICKNESS AbH R	1.123 ± 0.091 (0.910–1.493) ^c^	1.052 ± 0.074 (0.867–1.231) ^a^	**<0.001 ^d^**	-	−0.430
AR THICKNESS FDB R	1.171 ± 0.106 (1.010–1.418) ^a^	1.043 ± 0.096 (0.912–1.464) ^c^	**<0.001 ^d^**	-	−0.521
AR THICKNESS QP R	1.232 ± 0.152 (1.016–1.721) ^a^	1.033 ± 0.071 (0.966–1.210) ^c^	**<0.001 ^d^**	-	−0.721
Left side					
Cross-Sectional Area					
FHB (resting) (cm^2^)	2.940 ± 0.666 (1.386–4.156) ^a^	2.748 ± 0.779 (1.005–4.377) ^a^	0.240 ^b^	0.264	-
FHB (contracted) (cm^2^)	3.432 ± 0.744 (1.488–4.994) ^a^	2.876 ± 0.802 (0.991–4.401) ^a^	**0.002 ^b^**	0.717	-
AbH (resting) (cm^2^)	2.409 ± 0.585 (1.206–3.500) ^a^	2.527 ± 0.789 (1139–4.711) ^a^	0.450 ^b^	−0.170	-
AbH (contracted) (cm^2^)	2.746 ± 0.624 (1.490–4.463) ^a^	2.584 ± 0.814 (1.220–4.693) ^a^	0.324 ^b^	0.222	-
FDB (resting) (cm^2^)	2.551 ± 0.482 (1.834–4.235) ^a^	2.202 ± 0.649 (1.331–3.919) ^a^	**0.004 ^b^**	0.610	-
QP (resting) (cm^2^)	1.756 ± 0.511 (0.798–3.072) ^a^	1.768 ± 0.467 (0.855–2.139) ^a^	0.911 ^b^	−0.025	-
QP (contracted) (cm^2^)	2.086 ± 0.575 (1.212–3.603) ^a^	1.900 ± 0.457 (1.055–3.045) ^a^	0.113 ^b^	0.359	-
AR CSA FHB L	1.174 ± 0.105 (0.995–1.502) ^a^	1.050 ± 0.090 (0.833–1.239) ^a^	**<0.001 ^b^**	1.324	-
AR CSA AbH L	1.151 ± 0.122 (0.995–1.575) ^a^	1.023 ± 0.054 (0.881–1.141) ^a^	**<0.001 ^b^**	1.344	-
AR CSA FDB L	1.093 ± 0.065 (0.965–1.249) ^a^	1.030 ± 0.114 (0.846–1.656) ^c^	**0.004 ^d^**	-	−0.320
AR CSA QP L	1.193 ± 0.166 (0.993–1.845) ^c^	1.030 ± 0.180 (0.772–1.796) ^c^	**<0.001 ^d^**	-	−0.450
Thickness					
FHB (resting) (cm)	1.249 ± 0.163 (0.789–1.612) ^a^	1.155 ± 0.236 (0.652–1.770) ^a^	**0.022 ^b^**	0.457	-
FHB (contracted) (cm)	1.527 ± 0.276 (1.276–2.017) ^c^	1.247 ± 0.244 (0.856–2.004) ^a^	**<0.001 ^d^**	-	−0.635
AbH (resting) (cm)	1.081 ± 0.207 (0.585–1.425) ^c^	1.091 ± 0.206 (0.666–1.588) ^a^	0.814 ^d^	-	−0.026
AbH (contracted) (cm)	1.247 ± 0.165 (0.834–1.514) ^a^	1.134 ± 0.209 (0.811–1.645) ^a^	**0.009 ^b^**	0.600	-
FDB (resting) (cm)	1.043 ± 0.192 (0.721–1.422) ^a^	0.830 ± 0.213 (0.567–2.126) ^c^	**<0.001 ^d^**	-	−0.426
FDB (contracted) (cm)	1.163 ± 0.247 (0.726–1.625) ^a^	0.864 ± 0.292 (0.580–1.396) ^c^	**<0.001 ^d^**	-	−0.495
QP (resting) (cm)	1.008 ± 0.201 (0.618–1.504) ^a^	1.036 ± 0.225 (0.579–1.657) ^a^	0.279 ^b^	−0.131	-
QP (contracted) (cm)	1.222 ± 0.220 (0.708–1.689) ^a^	1.062 ± 0.225 (0.585–1.738) ^a^	**<0.001 ^b^**	0.717	-
AR THICKNESS FHB L	1.245 ± 0.097 (1.042–2.008) ^c^	1.085 ± 0.089 (0.928–1.368) ^a^	**<0.001 ^d^**	-	−0.702
AR THICKNESS AbH L	1.095 ± 0.158 (0.972–2.164) ^c^	1.043 ± 0.073 (0.916–1.248) ^a^	**<0.001 ^d^**	-	−0.495
AR THICKNESS FDB L	1.095 ± 0.070 (0.978–1.328) ^c^	1.031 ± 0.066 (0.906–1.214) ^a^	**<0.001 ^d^**	-	−0.446
AR THICKNESS QP L	1.198 ± 0.136 (0.919–1.939) ^c^	1.015 ± 0.049 (0.939–1.164) ^c^	**<0.001 ^d^**	-	−0.751

Abbreviations: FHB, flexor hallucis brevis; AbH, abductor hallucis; FDB, flexor digitorum brevis; QP, quadratus plantae; AR, activation range; R, right side; L, left side. ^a^ Mean ± standard deviation (SD) (minimum-maximum). ^b^ Student’s *t*-test for independent samples. ^c^ Median ± interquartile range (IR) (minimum-maximum). ^d^ Wilcoxon Mann–Whitney *U* test.

**Table 3:** Ultrasound imaging measurements and activation ranges of PF, CFP, and AT.

Measurement	Control Group (n = 40)	Case Group (n = 40)	*p*-Value	d-Cohen	r (Rosenthal)
Right side					
Thickness					
PF (resting) (cm)	0.317 ± 0.072 (0.208–0.508) ^c^	0.425 ± 0.087 (0.287–0.719) ^a^	**<0.001 ^d^**	-	−0.564
PF (contracted) (cm)	0.325 ± 0072 (0.200–0.492) ^c^	0.328 ± 0.086 (0.182–0.589) ^a^	0.832 ^d^	-	−0.024
CFP (resting) (cm)	0.800 ± 0.156 (0.515–1.221) ^a^	0.687 ± 0.173 (0.369–1.128) ^a^	**0.003 ^b^**	0.684	-
CFP (contracted) (cm)	0.816 ± 0.167 (0.511–1.313) ^a^	0.699 ± 0.174 (0.434–1.051) ^a^	**0.003 ^b^**	0.689	-
AT (resting)(cm)	0.514 ± 0.076 (0.361–0.701) ^a^	0.542 ± 0.087 (0.310–0.717) ^a^	0.125 ^b^	−0.347	-
AT (contracted)(cm)	0.533 ± 0.087 (0.333–0.732) ^a^	0.570 ± 0.104 (0.314–0.771) ^a^	0.090 ^b^	−0.384	-
AR THICKNESS PF R	0.979 ± 0.076 (0.868–1.293) ^c^	0.780 ± 0160 (0.456–1.181) ^a^	**<0.001 ^d^**	-	−0.645
AR THICKNESS CFP R	1.020 ± 0.086 (0.859–1.266) ^c^	1.018 ± 0.135 (0.861–1.625) ^c^	0.715 ^d^	-	−0.041
AR THICKNESS AT R	1.038 ± 0.098 (0.892–1.293) ^a^	1.060 ± 0.168 (0.744–1.352) ^a^	0.476 ^b^	−0.160	-
Left side					
Thickness					
PF (resting) (cm)	0.336 ± 0.063 (0.232–0.479) ^a^	0.381 ± 0.153 (0.205–0.689) ^c^	**<0.001 ^d^**	-	−0.391
PF (contracted) (cm)	0.331 ± 0.074 (0.196–0.481) ^a^	0.290 ± 0.129 (0.197–0.556) ^c^	0.305 ^d^	-	−0.115
CFP (resting) (cm)	0.830 ± 0.172 (0.542–1.415) ^a^	0.725 ± 0.180 (0.449–1.208) ^a^	**0.009 ^b^**	0.599	-
CFP (contracted) (cm)	0.817 ± 0.152 (0.567–1.372) ^a^	0.687 ± 0.192 (0.396–1.246) ^c^	**0.001 ^d^**	-	−0.366
AT (resting) (cm)	0.527 ± 0.064 (0.350–0.682) ^a^	0.545 ± 0.070 (0.404–0.721) ^a^	0.246 ^b^	−0.262	-
AT (contracted)(cm)	0.550 ± 0.078 (0.376–0.770) ^a^	0.590 ± 0.108 (0.379–0.863) ^a^	0.067 ^b^	−0.416	-
AR THICKNESS PF L	0.997 ± 0.112 (0.793–1.138) ^c^	0.789 ± 0.121 (0.566–1.103) ^a^	**<0.001 ^d^**	-	−0.681
AR THICKNESS CFP L	0.988 ± 0.069 (0.757–1.227) ^c^	1.011 ± 0.196 (0.664–1.247) ^c^	0.700 ^d^	-	−0.043
AR THICKNESS AT L	1.043 ± 0.068 (0.889–1.171) ^a^	1.100 ± 0.212 (0.734–1.403) ^c^	**0.036 ^d^**	-	−0.235

Abbreviations: PF, plantar fascia; CFP, calcaneal fat pad; AT, Achilles tendon; AR, activation range. ^a^ Mean ± standard deviation (SD) (minimum-maximum). ^b^ Student’s *t*-test for independent samples. ^c^ Median ± interquartile range (IR) (minimum-maximum). ^d^ Wilcoxon Mann–Whitney *U* test.

**Table 4:** Correlation coefficients (Pearson and Spearman’s rank correlations) for FHB, AbH, FDB, QP, PF, CFP, and AT in the right versus the left foot.

Measurement	Right Foot (N = 80)	Left Foot (N = 80)	*p*-Value	Pearson’s r	Spearman’s ρ
Resting state					
Cross-Sectional Area					
FHB (cm^2^)	2.717 ± 0.793 (0.831–4.445) ^a^	2.844 ± 0.726 (1.005–4.377) ^a^	**<0.001 ^‡^**	0.630	-
AbH (cm^2^)	2.514 ± 0.554 (0.888–3.950) ^a^	2.246 ± 0.692 (1.139–3.572) ^a^	**<0.001 ^‡^**	0.685	-
FDB (cm^2^)	2.344 ± 0.594 (1.343–4.185) ^a^	2.377 ± 0.595 (1.331–4.235) ^a^	**<0.001 ^‡^**	0.786	-
QP (cm^2^)	1.736 ± 0.407 (0.745–2.783) ^a^	1.650 ± 0.487 (0.798–3.139) ^b^	**<0.001 ^†^**	-	0.538
Thickness					
FHB (cm)	1.181 ± 0.208 (0.654–1.671) ^a^	1.201 ± 0.207 (0.652–1.770) ^a^	**<0.001 ^‡^**	0.625	-
AbH (cm)	1.090 ± 0.188 (0.758–1.695) ^b^	1.087 ± 0.190 (0.585–1.588) ^a^	**<0.001 ^†^**	-	0.578
FDB (cm)	0.913 ± 0.157 (0.545–1.261) ^a^	0.971 ± 0.233 (0.567–2.126) ^a^	**<0.001 ^‡^**	0.588	-
QP (cm)	1.038 ± 0.192 (0.478–1.538) ^a^	1.022 ± 0.213 (0.579–1.657) ^a^	**<0.001 ^‡^**	0.513	-
PF (cm)	0.377 ± 0.119 (0.208–0.719) ^b^	0.358 ± 0.095 (0.205–0.689) ^b^	**<0.001 ^†^**	-	0.653
CFP (cm)	0.743 ± 0.173 (0.369–1.221) ^a^	0.777 ± 0.183 (0.449–1.415) ^a^	**<0.001 ^‡^**	0.649	-
AT (cm)	0.528 ± 0.083 (0.310–0.717) ^a^	0.536 ± 0.067 (0.350–0.721) ^a^	**<0.001 ^‡^**	0.584	-
Contraction state					
Cross-Sectional Area					
FHB (cm^2^)	3.042 ± 0.885 (0.943–4.599) ^a^	3.154 ± 0.818 (0.991–4.994) ^a^	**<0.001 ^‡^**	0.652	-
AbH (cm^2^)	2.733 ± 0.621 (1.065–4.464) ^a^	2.665 ± 0.725 (1.220–4.693) ^a^	**<0.001 ^‡^**	0.717	-
FDB (cm^2^)	2.498 ± 0.653 (1.390–4.411) ^a^	2.557 ± 0.674 (1.278–4.469) ^a^	**<0.001 ^‡^**	0.741	-
QP (cm^2^)	1.957 ± 0.514 (1.005–3.697) ^a^	1.993 ± 0.525 (1.055–3.603) ^a^	**<0.001 ^‡^**	0.604	-
Thickness					
FHB (cm)	1.355 ± 0.256 (0.706–1.891) ^a^	1.404 ± 0.264 (0.856–2.017) ^a^	**<0.001 ^‡^**	0.702	-
AbH (cm)	1.211 ± 0.191 (0.758–1.785) ^a^	1.190 ± 0.195 (0.811–1.645) ^a^	**<0.001 ^‡^**	0.667	-
FDB (cm)	1.019 ± 0.201 (0.661–1.503) ^a^	1.034 ± 0.387 (0.580–1.625) ^b^	**<0.001 ^†^**	-	0.806
QP (cm)	1.170 ± 0.201 (0.763–1.707) ^a^	1.142 ± 0.235 (0.585–1.738) ^a^	**<0.001 ^‡^**	0.623	-
PF (cm)	0.317 ± 0.092 (0.182–0.589) ^b^	0.313 ± 0.113 (0.196–0.556) ^b^	**<0.001 ^†^**	-	0.495
CFP (cm)	0.758 ± 0.179 (0.434–1.313) ^a^	0.760 ± 0.168 (0.396–1.372) ^a^	**<0.001 ^‡^**	0.585	-
AT (cm)	0.551 ± 0.097 (0.314–0.771) ^a^	0.570 ± 0.095 (0.376–0.863) ^a^	**<0.001 ^‡^**	0.654	-

Abbreviations: FHB, flexor hallucis brevis; AbH, abductor hallucis; FDB, flexor digitorum brevis; QP, quadratus plantae; PF: plantar fascia; CFP: calcaneal fat pad; AT: Achilles tendon. ^a^ Mean ± standard deviation (SD) (minimum-maximum). ^b^ Median ± interquartile range (IR) (minimum–maximum). ^‡^ Pearson’s correlation coefficient. ^†^ Spearman’s rank correlation coefficient.

**Table 5:** Comparisons for CSA and thickness of FHB, AbH, FDB, QP, PF, CFP, and AT between the right and left foot in basketball players.

Measurement	Right foot (N = 80)	Left foot (N = 80)	*p*-Value	d-Cohen	r (Rosenthal)
Resting state					
Cross-Sectional Area					
FHB (cm^2^)	2.717 ± 0.793 (0.831–4.445) ^a^	2.844 ± 0.726 (1.005–4.377) ^a^	0.087 ^‡^	−0.194	-
AbH (cm^2^)	2.514 ± 0.554 (0.888–3.950) ^a^	2.246 ± 0.692 (1.139–3.572) ^a^	0.420 ^‡^	0.091	-
FDB (cm^2^)	2.344 ± 0.594 (1.343–4.185) ^a^	2.377 ± 0.595 (1.331–4.235) ^a^	0.461 ^‡^	−0.083	-
QP (cm^2^)	1.736 ± 0.407 (0.745–2.783) ^a^	1.650 ± 0.487 (0.798–3.139) ^b^	0.436 ^†^	-	−0.087
Thickness					
FHB (cm)	1.181 ± 0.208 (0.654–1.671) ^a^	1.201 ± 0.207 (0.652–1.770) ^a^	0.331 ^‡^	−0.114	-
AbH (cm)	1.090 ± 0.188 (0.758–1.695) ^b^	1.087 ± 0.190 (0.585–1.588) ^a^	0.561 ^†^	-	−0.065
FDB (cm)	0.913 ± 0.157 (0.545–1.261) ^a^	0.971 ± 0.233 (0.567–2.126) ^a^	0.008 ^‡^	−0.306	-
QP (cm)	1.038 ± 0.192 (0.478–1.538) ^a^	1.022 ± 0.213 (0.579–1.657) ^a^	0.488	0.078	-
PF (cm)	0.377 ± 0.119 (0.208–0.719) ^b^	0.358 ± 0.095 (0.205–0.689) ^b^	0.816 ^†^	-	−0.026
CFP (cm)	0.743 ± 0.173 (0.369–1.221) ^a^	0.777 ± 0.183 (0.449–1.415) ^a^	**0.044 ^‡^**	−0.229	-
AT (cm)	0.528 ± 0.083 (0.310–0.717) ^a^	0.536 ± 0.067 (0.350–0.721) ^a^	0.323 ^‡^	−0.111	-
Contraction state					
Cross-Sectional Area					
FHB (cm^2^)	3.042 ± 0.885 (0.943–4.599) ^a^	3.154 ± 0.818 (0.991–4.994) ^a^	0.165 ^‡^	−0.157	-
AbH (cm^2^)	2.733 ± 0.621 (1.065–4.464) ^a^	2.665 ± 0.725 (1.220–4.693) ^a^	0.242 ^‡^	0.132	-
FDB (cm^2^)	2.498 ± 0.653 (1.390–4.411) ^a^	2.557 ± 0.674 (1.278–4.469) ^a^	0.274 ^‡^	−0.123	-
QP (cm^2^)	1.957 ± 0.514 (1.005–3.697) ^a^	1.993 ± 0.525 (1.055–3.603) ^a^	0.485 ^‡^	−0.078	-
Thickness					
FHB (cm)	1.355 ± 0.256 (0.706–1.891) ^a^	1.404 ± 0.264 (0.856–2.017) ^a^	**0.033 ^‡^**	−0.243	-
AbH (cm)	1.211 ± 0.191 (0.758–1.785) ^a^	1.190 ± 0.195 (0.811–1.645) ^a^	0.252 ^‡^	0.129	-
FDB (cm)	1.019 ± 0.201 (0.661–1.503) ^a^	1.034 ± 0.387 (0.580–1.625) ^b^	0.238 ^†^	-	−0.132
QP (cm)	1.170 ± 0.201 (0.763–1.707) ^a^	1.142 ± 0.235 (0.585–1.738) ^a^	0.104 ^‡^	0.142	-
PF (cm)	0.317 ± 0.092 (0.182–0.589) ^b^	0.313 ± 0.113 (0.196–0.556) ^b^	0.866 ^†^	-	−0.019
CFP (cm)	0.758 ± 0.179 (0.434–1.313) ^a^	0.760 ± 0.168 (0.396–1.372) ^a^	0.917 ^‡^	−0.012	-
AT (cm)	0.551 ± 0.097 (0.314–0.771) ^a^	0.570 ± 0.095 (0.376–0.863) ^a^	**0.041 ^‡^**	−0.232	-

Abbreviations: FHB, flexor hallucis brevis; AbH, abductor hallucis; FDB, flexor digitorum brevis; QP, quadratus plantae; PF: plantar fascia; CFP: calcaneal fat pad; AT: Achilles tendon. ^a^ Mean ± standard deviation (SD) (minimum–maximum). ^b^ Median ± interquartile range (IR) (minimum–maximum). ^‡^ Student’s *t*-test for related samples. ^†^ Wilcoxon test for related samples.

## Data Availability

The authors have no competing interests to declare that are relevant to the content of this article.
